# Predictive and prognostic biomarkers of Bacillus Calmette‐Guérin therapy failure in bladder cancer patients: A systematic review

**DOI:** 10.1002/1878-0261.70329

**Published:** 2026-09-07

**Authors:** Rui Ribeiro‐Pereira, Ana Teixeira‐Marques, Rui Freitas, João A Carvalho, Sara Monteiro‐Reis, Giorgio I. Russo, Rui Henrique, Carmen Jerónimo

**Affiliations:** ^1^ Cancer Biology and Epigenetics Group IPO Porto Research Center, Portuguese Oncology Institute of Porto (IPO Porto) Porto Portugal; ^2^ RISE – Associate Laboratory (Health Research Network) Porto Portugal; ^3^ Porto.CCC – Porto Comprehensive Cancer Centre Raquel Seruca Porto Portugal; ^4^ Master Programme in Oncology, ICBAS‐School of Medicine and Biomedical Sciences University of Porto Porto Portugal; ^5^ Doctoral Programme in Biomedical Sciences, ICBAS‐School of Medicine and Biomedical Sciences University of Porto Porto Portugal; ^6^ Department of Urology Portuguese Oncology Institute of Porto (IPO Porto) Porto Portugal; ^7^ Urology Section, Department of Surgery University of Catania Catania Italy; ^8^ Department of Pathology Portuguese Oncology Institute of Porto (IPO Porto) Porto Portugal; ^9^ Department of Pathology and Molecular Immunology, ICBAS‐School of Medicine and Biomedical Sciences University of Porto Porto Portugal

**Keywords:** BCG therapy, Bladder Cancer, molecular marker, predictive biomarkers, PRISMA, prognosis biomarkers

## Abstract

High‐risk non‐muscle invasive bladder cancer (NMIBC) is a common urological malignancy with a high propensity for recurrence (30–40% of patients) despite treatment with Bacillus Calmette–Guérin (BCG) instillations. Identifying patients who are likely to respond or not to BCG therapy before treatment initiation may significantly improve patient outcomes and minimise unnecessary treatments. This systematic review critically assessed the existing literature on the potential of non‐invasive biomarkers to predict BCG failure in NMIBC patients. A comprehensive bibliographic search on PubMed, Scopus and Medline databases was conducted to identify relevant studies published up to July 7, 2025. After a thorough screening and selection process, 72 studies were included in the final analysis, 58 of which focused on prognostic markers, 6 on predictive markers, and 8 examined both. Moreover, 55 studies focused on tissue, while 28 studied liquid biopsy‐derived biomarkers, showcasing their ability to reflect the dynamic characteristics of the tumour and its microenvironment. The most frequently studied biomarkers comprised p53, Ki‐67 and pRB. Furthermore, several immunological markers were also explored, highlighting the current challenge of BCG therapy prediction biomarkers. Despite its valuable insights, this systematic review faced limitations within the included studies, such as issues related to reproducibility, feasibility, and small sample sizes. Nonetheless, the findings indicate that a wide array of molecules show potential as prognostic and/or predictive markers for BCG failure, which may improve the clinical management of this complex disease.

## Graphical abstract

High‐risk bladder cancer is typically treated with Bacillus Calmette‐Guérin (BCG), but 30–40% of patients relapse. No FDA‐ or CE‐approved biomarkers currently predict or prognosticate BCG failure. We systematically reviewed the literature and identified 72 eligible studies, revealing several promising biomarkers associated with BCG treatment response and clinical outcomes.

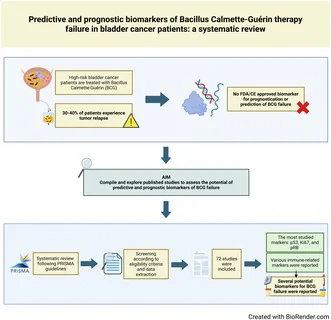

**Abbreviations**
A1AT: alpha‐1 antitrypsin
ANG: angiogenin
APOE: apolipoprotein E
AUA: American Urological Association
AUC: area under the curve
BCG: Bacillus Calmette‐Guérin
BlCa: bladder cancer
C12orf42: chromosome 12 open frame 42
CA9: carbonic anhydrase 9
CCL27: C‐C motif chemokine ligand 27
CD: clusters of differentiation
CE: Conformité Européene
CHST11: carbohydrate sulfotransferase 11
Cis: carcinoma *in situ*
CSS: cancer‐specific survival
CTLA‐4: cytotoxic T‐lymphocyte‐associated protein 4
DNA: deoxyribonucleic acid
DSS: disease‐specific survival
EAU: European Association of Urology
EFS: event‐free survival
FASL: FAS ligand
FDA: food and drug administration
FFPE: formalin‐fixed paraffin embedded
FLT1: vascular endothelial growth factor receptor 1
FOXP3: forkhead box P3
G1: grade 1
G2: grade 2
G3: grade 3
GPR58: G protein‐coupled receptor 58
HG: high‐grade
HR: hazard ratio
iBCG: induction Bacillus Calmette‐Guérin scheme
IL: interleukin
Ki‐67: kiel 67
KLF8: krüppel‐like factor 8
LG: low‐grade
mBCG: maintenance Bacillus Calmette‐Guérin scheme.
MIBC: muscle invasive bladder cancer
MITc: mitomycin C
MMP10: matrix metallopeptidase 10
MMP9: matrix metallopeptidase 9
NMIBC: non‐muscle invasive bladder cancer
OR: odds ratio
OS: overall survival
P53: protein 53
PAI1: plasminogen activator inhibitor 1
PBL: peripheral blood leucocyte
PCR: polymerase chain reaction
PD‐1: programmed cell death protein 1
PFS: progression‐free survival
pRB: retinoblastoma protein
PRISMA: preferred reporting items for systematic reviews and meta‐Analyses.
PROBAST: prediction model risk of bias assessment.
PROSPERO: international prospective register of systematic reviews
QUAPAS: quality assessment of prognostic accuracy studies.
RFS: recurrence‐free survival
RNA: ribonucleic acid
ROC: receiver operating characteristic
SDC1: syndecan‐1
TNF‐α: tumour necrosis factor‐alpha
TRAIL: tumour necrosis factor‐related apoptosis‐inducing ligand
TURBT: transurethral resection of the bladder tumour
VEGFA: vascular endothelial growth factor A
WDR44: WD repeat‐containing protein 44
yr: year

## 1 Introduction

### 1.1 Bladder cancer epidemiology

Bladder cancer (BlCa) is the 9^th^ most common malignancy diagnosed worldwide, with approximately 614 000 new cases and 220 000 deaths estimated in 2022 [1]. Although BlCa ranks among the most expensive cancers to treat over a lifetime, public policies and research are considerably lagging behind the societal relevance of the disease and its impact on patients and their caregivers, healthcare systems and economies, being, thus, regarded as the ‘Cinderella’ of cancers [2, 3].

Most types of bladder tumours originate in the urothelium, with urothelial carcinoma constituting the most frequent BlCa type, comprising 90% of all diagnosed cases [4]. Clinically, this malignancy is usually stratified into superficial, non‐muscle invasive bladder cancer (NMIBC), comprising 75–80% of cases, and muscle‐invasive bladder cancer (MIBC), accounting for the remainder [4].

### 1.2 Bladder cancer management and treatment: A brief overview

Most BlCa cases are diagnosed following cystoscopy examination performed due to haematuria and/or other suspicious lower urinary tract symptoms, complemented with excision of visible lesions through transurethral resection of bladder tumour (TURBT) [5]. Final diagnosis requires histopathologic evaluation of the tissue obtained [5]. Thereafter, patients are generally stratified according to the European Association of Urology (EAU) or the American Urological Association (AUA) prognostic groups, based on the histopathologic features of the tumour, multifocality, stage, size, previous recurrences and concomitant urothelial carcinoma *in situ* (Cis) [6, 7]. Risk stratification is key to decide on the most appropriate clinical management, including adjuvant treatment options and follow‐up strategy, depending on the probability of recurrence and/or progression [6, 7]. Thus, intermediate‐risk patients are often treated with intravesical chemotherapy (e.g. mitomycin C) to prevent recurrences [8]. Bacillus Calmette–Guérin (BCG) instillation is also an option for these patients, although they may develop more therapy‐related side effects compared to those submitted to intravesical chemotherapy [6, 7, 9]. High‐risk patients, on the other hand, are usually treated with BCG, which was proven to delay disease recurrence and progression [6, 7].

### 1.3 Limitations of Bacillus Calmette‐Guérin treatment management

Although prognostic tables allow for stratification of patients for treatment guidance, these tools fail to adequately portray the molecular heterogeneity and complexity of BlCa, consequently leading to a high rate of treatment failure, especially relapse. Although BCG prevents progressions and recurrences, a high risk of relapse remains (30–40%) [10, 11]. Currently, there is no Food and Drug Administration or Conformité Européenne (FDA/CE) approved biomarker to prospectively discriminate among patients that will positively respond to BCG from those which will fail, resulting in significantly increased morbidity and economic burden on healthcare systems. Hence, identification of predictive and prognostic biomarkers for BCG immunotherapy failure might considerably improve patient outcome and avoid ineffective treatment.

Considering the lack of approved biomarkers predictive of BCG failure to assist therapy decisions, we performed a systematic review, compiling and exploring published studies to assess the potential of predictive and prognostic biomarkers of BCG failure in BlCa patients. We expect that a critical analysis of published literature may disclose new avenues for the development of clinically useful tools in this setting.

## 2 Methods

### 2.1 Protocol and registration

This systematic review followed the most recent guidelines of the preferred reporting items for systematic reviews and meta‐analysis (PRISMA) [12]. The protocol for the study was registered after data extraction with the international prospective register of systematic reviews (PROSPERO) and assigned the registration ID CRD42024560005.

### 2.2 Retrieval methodology

To perform this comprehensive systematic review, an extensive search was conducted across three major scientific databases: PubMed, Scopus and Medline. The search aimed to identify all published studies investigating the potential of different molecules as prognostic and/or predictive biomarkers of failure to BCG treatment in patients with BlCa. The search was limited to studies published until July 7, 2025.

In the search for the most relevant studies, various combinations of terms were created, including ‘Bladder Cancer’, ‘Biomarker’ and ‘BCG’, which were utilised to construct queries for searching in different databases. The query used for search in PubMed was the following: (((“Urinary Bladder Neoplasms”[MeSH Terms]) OR (“Non‐muscle invasive bladder neoplasms”[MeSH Terms]) OR (“bladder cancer”[title/abstract]) OR (“bladder urothelial carcinoma”[title/abstract]) OR (“lower tract urothelial carcinoma”[title/abstract]) OR (“Papillary urothelial carcinoma”[title/abstract]) OR (“Transitional Cell Bladder Cancer”[title/abstract])) AND ((“Biomarkers”[MeSH Terms]) OR (“Biomarkers, Tumor”[MeSH Terms]) OR (“Biomarker”[title/abstract]) OR (“Molecular Marker”[title/abstract]) OR (“Marker”[title/abstract])) AND ((“BCG”[title/abstract]) OR (“Bacillus Calmette‐Guérin”[title/abstract]) OR (“Immunotherapy”[title/abstract]) OR (“Immunotherapy”[MeSH Terms]))). The query used for search in Scopus was the following: TITLE‐ABS‐KEY(((“Urinary Bladder Neoplasms”) OR (“Non‐muscle invasive bladder neoplasms”) OR (“bladder cancer”) OR (“bladder urothelial carcinoma”) OR (“lower tract urothelial carcinoma”) OR (“Papillary urothelial carcinoma”) OR (“Transitional Cell Bladder Cancer”)) AND ((“Biomarkers”) OR (“Biomarker”) OR (“Molecular Marker”) OR (“Marker”)) AND ((“BCG”) OR (“Bacillus Calmette‐Guérin”))). The query used for search in Medline was the following: (((MH Urinary Bladder Neoplasms) OR (MH Non‐muscle invasive bladder neoplasms) OR (AB bladder cancer) OR (AB bladder urothelial carcinoma) OR (AB lower tract urothelial carcinoma) OR (AB Papillary urothelial carcinoma) OR (AB Transitional Cell Bladder Cancer)) AND ((MH Biomarkers) OR (MH Biomarkers, Tumor) OR (AB Biomarker) OR (AB Molecular Marker) OR (AB Marker)) AND ((AB BCG) OR (AB Bacillus Calmette‐Guérin))).

### 2.3 Eligibility criteria

Following the elimination of duplicate records, the resulting publications underwent a rigorous review process based on their title and abstract, adhering to a set of predetermined eligibility criteria established by 2 independent authors (RR‐P and AT‐M) who worked in a blinded manner.

To conduct this systematic review, inclusion criteria encompassed molecular biomarker studies in patients with lower tract urothelial carcinoma treated with BCG immunotherapy, focusing on patients' prognosis and/or prediction of failure to treatment. Additionally, papers assessing prognostic and predictive molecular biomarkers with respective survival and/or performance analysis in patients who underwent BCG were included.

Regarding exclusion criteria, it was decided that studies that focused only on types of BlCa other than NMIBC would be excluded. The same was applied to publications performed only on *in vivo/in vitro* models, records that did not reach statistical significance, and studies that were not original, such as editorials, letters, case reports, meta‐analyses, and other reviews. Studies published in languages other than English or Portuguese were also excluded from the analysis.

### 2.4 Data extraction

During the evaluation process, the remaining studies underwent a thorough assessment to determine their eligibility for further analysis. Thus, 2 blinded independent authors extracted the relevant information from the selected records. The extracted information for both prognostic and predictive studies included the size of the cohort of interest, tumour stage, histological grade, treatment scheme, outcome (as reported by the authors in the article), source of the material used, type of molecule, and respective expression level. Additionally, for prognostic studies, survival analysis, clinical significance, and hazard ratio (HR) or odds ratio (OR) were mentioned, while for predictive studies, the biomarker performance parameters (sensitivity, specificity, and area under the curve (AUC)) were recorded when available.

### 2.5 Risk of bias assessment

#### 2.5.1 Prognostic studies

To evaluate the quality of prognostic studies, the adapted quality assessment of prognostic accuracy studies (QUAPAS) tool was utilised [13]. Assessment of risk of bias included participants, index test, outcome, flow and timing, and analysis evaluation. Moreover, 4 domains, namely participants, index test, outcome, and flow and timing, were also assessed for concerns regarding applicability.

#### 2.5.2 Predictive studies

To conduct a more thorough quality assessment of the selected predictive studies, we utilised adapted available criteria from the prediction model risk of bias assessment tool (PROBAST) [14]. Thus, 5 key domains were covered: participants, predictors, outcome, flow and timing, and analysis. Concerns regarding applicability included participants, predictors, outcome, and flow and timing.

## 3 Results

### 3.1 Literature overview

**Fig. 1:**
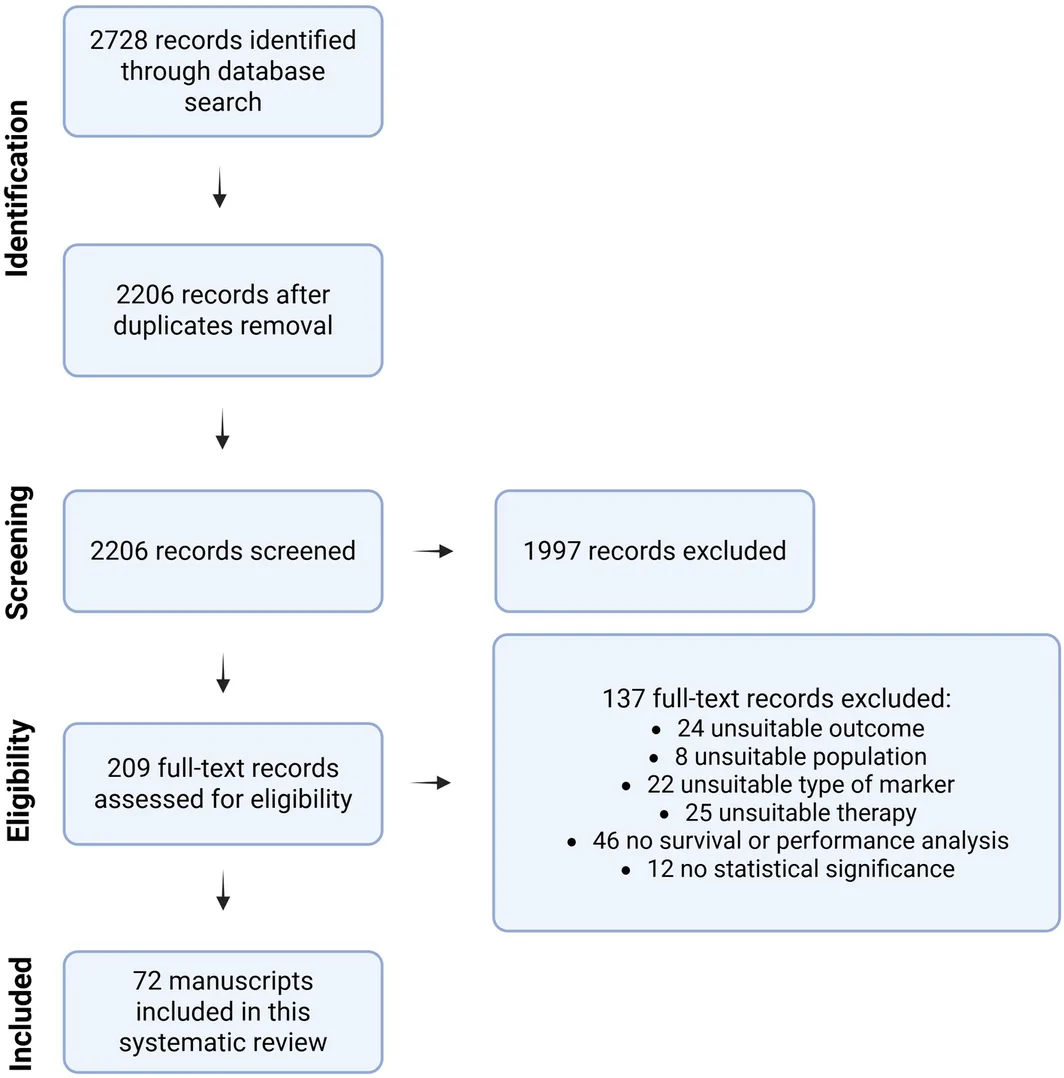
Flowchart of the literature selection process for the included records, according to the preferred reporting items for systematic reviews and meta‐analyses (PRISMA) guidelines.

We identified a total of 2328 records through bibliographic search, which were sourced from three distinct public databases: PubMed (1324), Scopus (983), and Medline (421). Out of these records, 522 were identified as duplicates. After a comprehensive review of titles and abstracts, we screened 2206 articles, excluding 1997 and retaining 209 for further analysis. Subsequently, 137 publications were excluded based on specific criteria: 24 manuscripts did not address BlCa prognostic and/or predictive of failure parameters, 8 focused on subtypes other than NMIBC, 22 were an unsuitable type of marker, 25 were excluded due to the therapy scheme (BCG was not performed or was combined with other therapeutic agents), 46 publications did not conduct any survival or biomarker performance analysis, and 12 did not reach statistical significance on discriminating BCG failure and non‐failure groups or in the performed survival/receiver operating characteristic (ROC) curve analysis and were consequently excluded. Finally, 22 studies were excluded because they were not focused on molecular biomarkers. Hence, 72 articles were included in the final analysis. The flow diagram depicted in Fig. 1 offers a comprehensive insight into the criteria employed for the study selection process.

Thus, an extensive review was conducted, covering over 30 years of research on prognostic and predictive biomarkers for BlCa following BCG treatment. It included studies from 1987 to 2025, mostly originating from Asian and Western populations, but fairly representing a global perspective. From the selected studies, 58 solely conducted survival analysis on the target prognostic markers, 6 only evaluated markers' performance in predicting treatment failure, and 8 focused on both prognostic and predictive markers of failure to BCG. A comprehensive summary of each study's information may be found in Tables 1 and 2.

**Table 1:** Information regarding prognostic studies on bladder cancer patients treated with Bacillus Calmette‐Guérin. ↑, high levels; ↓, low levels; BCG, Bacillus Calmette‐Guérin; BlCa, bladder cancer; Cis, carcinoma *in situ*; CSS, cancer‐specific survival; DFS, disease‐free survival; DSS, disease‐specific survival; EFS, event‐free survival; G1, grade 1; G2, grade 2; G3, grade 3; HG, high‐grade; HR, hazard ratio; iBCG, induction BCG scheme; IG, intermediate grade; LG, low‐grade; mBCG, maintenance BCG scheme; MITc, mitomycin C; OR, Odds ratio; OS, overall survival; PBL, peripheral blood leukocyte; PFS, progression‐free survival; RFS, recurrence‐free survival; yr, years.

Study (ref)	Year	Cohort	Stage (*n*)	Histologic grade (*n*)	Outcome (*n*)	Treatment scheme	Molecule	Level of molecule	Clinical significance	Survival analyses: HR/OR
1. Tissue										
1.1 Protein										
X7 [50]	2025	20	–	–	–	–	CIT	↑ BCG Failure	RFS	1.35
X6 [51]	2025	73	Cis (73)	–	–	iBCG + mBCG	AR	↑ BCG Failure	RFS	2.52
							ADAR2			0.29
							PD‐L1			5.22
X5 [52]	2025	90	Cis (42), Concomitant Cis (25), Ta (18), T1 (30)	LG (5), HG (85)	–	iBCG + mBCG	NANOG	↑ BCG Failure	RFS	2.49
									PFS	3.92
X3 [53]	2025	77	Ta (13), T1 (64)	LG (74), HG (3)	BCG Unresponsive (39), BCG Responsive (38)	iBCG + mBCG	CD56	↑ BCG Response	RFS	1.83
V3 [54]	2024	40	Cis (1), Ta (5), T1 (34)	LG (5), IG (1), HG (34)	–	iBCG + mBCG	TERT C228T KDM6A	↑ Recurrence	RFS	–
V2 [55]	2024	174	T1 (91), T2 (68), T3/T4 (15)	HG (81), LG (93)	–	–	PRKG1	↓ Recurrence	RFS	–
V1 [56]	2024	106	Cis (3), Ta (20), T1 (83)	HG (85), LG (19)	Responder (47), Non‐Responders (59)	iBCG + mBCG	CD276 TIGIT	↑ BCG Non‐Response	–	–
							PD‐1 CTLA4 TNFRSF14	↑ BCG Response		
U4 [57]	2023	67	Ta (26), T1 (41)	HG (54), LG (10)	Recurrence (38), Progression (7)	iBCG	HER‐2	↑ Recurrence	RFS	2.686
U1 [58]	2023	454	Cis (21), Ta (215), T1 (218)	HG (403), LG (51)	Recurrence (89)	iBCG + mBCG	HER‐2	↑ BCG Failure	RFS	2.36
								–	PFS	2.91
Q1 [59]	2019	22	T1	HG	Responders (12), Non‐Responders (10)	iBCG + mBCG	Peritumoral T bet+/Lymphocyte	↑ Recurrence	RFS	5.3
							[GATA‐3+/T‐bet+]/[neutrophil/lymphocyte]	↓ Recurrence		0.18
							Peritumoral T bet+	↑ Recurrence	–	–
P2 [24]	2018	78	T1 (78), Concomitant Cis (18)	G1/2(30), G3(48)	–	iBCG	PD‐1	↑ BCG Failure	RFS	2.99
									PFS	8.27
P1 [60]	2018	54	T1	HG	Recurrence (25), Progression (14)	iBCG iBCG + mBCG	HSP60 > 65%	↑ BCG Failure	PFS	3.96
							HSP70 > 5%		PFS	0.33
								↑ BCG Response	RFS	0.29
O4 [61]	2017	41	Ta (12), T1 (28), Cis (1)	G2 (3), G3 (38)	–	iBCG + mBCG	IL‐17	↑ BCG Response	EFS	0.2918
O2 [62]	2017	34	T1	G3	Recurrence (18), Progression (17)	iBCG + mBCG	HER‐2	↓ Recurrence	DFS	2.7968
									PFS	2.8161
O3 [63]	2017	71	Cis, Ta, T1	LG, HG	Recurrence (40), Progression (17)	iBCG	Tregs (FOXP3+)	↑ BCG Failure	RFS	3.07
									PFS	3.43
							TAM (CD204+)		RFS	–
									PFS	–
N1 [22]	2016	40	Cis (10), Ta (9), T1 (21)	LG (7) and HG (33)	–	–	CD4/CD8 ratio	↓ BCG Failure	RFS	0.03
							CD68	↑ BCG Failure		3.34
							CD163	↑ BCG Failure		2.01
							CD163/CD68 ratio	↑ BCG Failure		2.38
							FOXP3	↑ BCG Failure		6.05
							CD25	↑ BCG Failure		4.27
							GATA3	↓ BCG Failure		0
							GATA3/T‐bet ratio	↓ BCG failure		0
							CD4+	↓ BCG failure		0.13
M1 [64]	2015	134	T1 (134), Concomitant Cis (78)	–	–	iBCG + mBCG	∆Np63	↑ Recurrence	OS	0.654
L1 [21]	2014	309	T1 + Concomitant Cis (106), T1 (202)	–	–	iBCG + mBCG	Ki‐67	↑ Progression	PFS	2.8
							CK20	↑ Recurrence	RFS	5.89
L2 [65]	2014	99	Ta (40), T1 (59), Concomitant Cis (7)	LG (39) and HG (60)	Responders (27), Non‐Responders (42)	iBCG + mBCG	CD163	↑ Recurrence	RFS	2.627
K2 [18]	2013	192	Ta (121), T1 (115), Concomitant Cis (10)	G1 (53), G2 (76), G3 (63)	Recurrence (111), Progression (20)	iBCG + mBCG	p53	↓ Recurrence	RFS	0.61
K4 [66]	2013	27	Ta (12), T1 (15)	LG (21), HG (6)	Responders (7), Non‐Responders (12)	iBCG + mBCG	CD68	↑ Recurrence	RFS	0.148
J3 [67]	2012	28	Ta (19), T1(9)	LG (19), HG (9)	–	iBCG	Bcl‐2	↑ Recurrence	RFS	3
							Bax			9.9
J4 [68]	2012	28	Ta (19), T1 (9)	LG (19), HG (9)	–	iBCG	CD34	↑ Recurrence	RFS	67.2
I3 [69]	2011	87	T1 (87)	G2 (15), G3 (72)	–	–	AQP3	↓ Progression	PFS	7.58
I4 [40]	2011	42	Ta (17), T1 (25), Concomitant Cis (8)	G1 (6), G2 (23) and G3 (13)	–	iBCG	MMP9, Bcl2, CD1, and p21	↑ Recurrence	RFS	–
							MMP9 and p21	↑ Progression	PFS	–
H2 [34]	2010	27	T1 (27)	G3 (27)	Recurrence (10), Progression (5)	iBCG + mBCG	pRB	–	PFS	–
									RFS	–
H3 [70]	2010	77	Ta (48), T1 (29)	G1 (35), G2 (28), G3 (14)	Recurrence (77)	iBCG + mBCG	INF‐α	Loss of Heterozygosity in Recurrence	RFS	4.09
G1 [35]	2009	27	T1 (27)	G3 (27)	Recurrence (10), Progression (5)	iBCG + mBCG	pRB and p53	–	PFS	–
G2 [71]	2009	92	T1 (91), Concomitant Cis (55)	G3 (91)	Recurrence (40), Progression (17)	iBCG	ezrin	↓ Progression and lower survival	DSS	–
G3 [72]	2009	66	Ta (30), T1 (23), Cis (13)	G1 (5), G2 (36), G3 (25)	–	iBCG + mBCG	CD68 expression and infiltration	↑ Recurrence	–	4.64
							CD68 expression		–	3.81
F1 [19]	2007	83	T1 (31), Concomitant Cis (12)	–	No progression (52), Progression (31)	iBCG + mBCG	p53	↑ Progression	PFS	6.44
E1 [17]	2004	102	T1a (53), T1b (17), Concomitant Cis (18)	G1‐G2 (64), G3 (38)	Recurrence (36), Progression (13), Without relapse (53)	iBCG	p53	↑ Recurrence	RFS	0.15
C2 [20]	1997	104	Ta (61), T1 (43)	G1 (30), G2 (63) and G3 (11)	Recurrence (60), Non‐Recurrence (44)	–	Ki‐67	↑ Recurrence	RFS	2.557
C1 [16]	1997	32	Papillary (26) Non‐papillary (6), Concomitant Cis (2)	G2 (7), G3 (8)	Responders (15), Non‐Responders (17)	iBCG	p53	↓ Non‐Responders	DFS	3.8
B1 [15]	1996	98	Ta (22), T1 (19), Cis (57)	–	Responders (62), Progression (29)	iBCG	p53	↓ Non‐Responders	PFS	6.7
1.2 DNA										
S1 [73]	2021	42	T1 (42)	HG (42)	Responders (17), Non‐Responders (25)	iBCG	*MLC1*	↑ BCG Response	RFS	0.77
R4 [74]	2020	35	Ta (10), T1 (25)	LG (5), HG (30)	Responders (17), Non‐Responders (18)	iBCG + mBCG	*TMB*	↑ BCG Response	RFS	0.28
							*NAL*			0.30
							*DDR*			0.27
R3 [75]	2020	125	Ta (51) T1 (74)	LG (40), HG (85)	–	iBCG + mBCG	*TERTp* c.1‐146G>A	↑ BCG Response	RFS	0.382
I2 [76]	2011	91	T1 (91)	G3 (91)	Recurrence (40), Progression (17)	iBCG	*MSH6, TP73*	↓ Progression	PFS	0.302
							*RB1, THBS1*			0.133
							*RB1, PYCARD*			0.149
							*THBS1, PYCARD*			0.211
							*MSH6, THBS1, PYCARD*			0.226
							*MSH6, THBS1, TP73*			0.226
							*RB1, THBS1, PYCARD*			0.134
							*MSH6, THBS1, RB1*			0.262
							*RB1, MSH6, TP73, THBS1*			0.226
							*MSH6, THBS1*			0.226
							*RB1, MSH6, TP73, THBS1, PYCARD*			0.226
H4 [77]	2010	108	–	–	Recurrence (35), Progression (21)	iBCG + mBCG	Myopodin	↑ Progression/Recurrence	RFS	2.541
									PFS	11.227
									DSS	7.552
H5 [78]	2010	28	Ta (6), T1 (15), T2 (4), Concomitant Cis (28)	G3 (28)	Cis Recurrence (9), Cis Progression (4)	iBCG + mBCG	Cyclin D3	Amplification in progression	PFS	61.503
1.3 RNA										
T5 [79]	2022	59	Ta (19), T1 (40)	LG (21) HG (38)	Recurrence (26), Non‐Recurrence (33)	–	*RPL9*	↓ BCG Failure	RFS	2.649
T3 [80]	2022	100	T1 (100)	G3 (100)	Responders (46), Non‐Responders (54)	iBCG + mBCG	miR‐31	Downregulated	↓ RFS	0.05
									↓ PFS	0.03
							miR‐let7a	Downregulated	↓ PFS	0.001
							miR‐199a	Upregulated	↓ RFS	2.8
							*ARID1A*	Downregulated	↓ RFS	0.9
									↓ PFS	0.18
							*NCOR1*	Upregulated	↓ RFS	0.9
							*UTX*	Downregulated	↓ RFS	0.03
							miR‐21	Upregulated	↓ PFS	1.2
							*STAG2*	Upregulated	↓ PFS	3.1
H1 [81]	2010	80	T1 (48)	G1 (6), G2 (33) and G3 (9)	Recurrence (22), Non‐Recurrence (26), Progression (5), No Progression (43)	iBCG	Recurrence: MAEA, SEC24C, ZC3HC1, RBAF600, AP1G1, UBE2I, HLA‐A, CEBPZ*, RNPS1, PTPRM, BTG2	↓ Recurrence/Progression, *↑ Recurrence/Progression	RFS	3.38
							Progression: VPS37C*, UFC1*, POLR2A, STOML2, RNF20, XRCC5, SMARC4A4*, SEPX1, SUGT1*, TAX1BP3, LGMN, HEBP2*		PFS	10.49
1.4 Glycan										
K3 [82]	2013	94	Ta (40), T1 (54), Concomitant Cis (6)	LG (38) and HG (56)	Recurrence (36)	iBCG + mBCG	sTn and/or s6T	↓ Recurrence	RFS	0.296
2. Tissue and Blood										
2.1. Protein										
R1 [23]	2020	29	Cis (2), Ta (11), T1 (16)	HG (5)	Responders (21), Non‐Responders (8)	iBCG	CD4 + FOXP3‐ T cells	↑ BCG Response	RFS	9.54
							CD8 + PD1+ T cells			9.96
2.2. DNA and RNA										
L3 [83]	2014	125	Ta (51), T1 (74), Concomitant Cis (13)	LG (26) and HG (99)	Responder (77), Non‐Responders (48)	iBCG iBCG + mBCG	*FASL*‐844 (TT + TC vs CC)	CC Genotype ↓RFS	RFS	1.922
							*FASL*	↑ Recurrence	RFS	2.833
3. Blood										
3.1 Protein										
U2 [84]	2023	1709	Cis (317), Ta (468), T1 (924)	LG (101), HG (1557), Not Evaluated (51)	–	iBCG iBCG + mBCG	C‐Reactive Protein	↑ BCG Failure	RFS	1.431
									PFS	1.660
									CSS	1.847
R2 [85]	2020	Discovery Cohort 13	T1 (50)	HG (9), LG (4)	Responders (9), Non‐Responders (4)	iBCG + mBCG	CCL27	–	–	–
		Validation Cohort 37		HG (24), LG (13)	Responders (28), Non‐Responders (9)			↑ BCG Response	RFS	4.650
N2 [86]	2016	1117 (MITc 48, BCG 300)	Ta (674), T1 (443), Concomitant Cis (66)	G1 (231), G2 (398), G3 (488)	–	iBCG	C‐Reactive Protein	↑ Progression	Disease progression	3.51
3.2 DNA										
X2 [87]	2025	151	Ta/Cis (56), T1 (117), T2 (2)	–	–	–	*HLA‐21 M/T*	↑ Progression	PFS	2.08
								–	OS	2.059
T1 [88]	2022	195	Ta (88), T1 (107)	G1 (10), G2 (44), G3 (140), NA (1)	Recurrence (43)	iBCG	*HLA*‐B62	↑ Recurrence	RFS	5.73
							*HLA‐*B07	↓ Recurrence	RFS	0.43
							*HLA‐*B044			0.36
M2 [89]	2015	BCG (51), Non‐treated (37)	Ta (16), T1 (63), Cis (9), Concomitant Cis (14)	G1/G2 (14) and G3 (28)	–	–	*NOS3* (TT vs CT/CC)	↓ Progression	PFS	0.05
							*NOS3* (GG vs GT/TT)	↓ Progression, Recurrence	RFS	0.29
									PFS	0.1
							*NOS2* promoter microsatellite (CCTTT)n ≧13 repeats)	–	CSD	0.12
M3 [37]	2015	204	Ta (82), T1 (122), Concomitant Cis (186)	LG (55) HG (149)	BCG Success (134), BCG Failure (70)	iBCG iBCG + mBCG	*TNF‐α*1031T/C (TT + TC vs CC)	CC genotype ↑ Recurrence	RFS	3.49
							*IL4*‐33 T/C (CT vs TT)	–		1.93
							*IL2RA* T/C (TT vs TC + CC)	TT genotype ↑ Recurrence		2.79
							*IL17A‐*197G/A (GG + GA vs AA)	AA genotype ↑ Recurrence		2.65
							*IL17RA*‐809A/G (AA+AG vs GG)	–		2.89
							*IL18R1* T/C (CC vs TC + TT)	–		2.75
							*FASL*‐844 T/C (TT + TC vs CC)	–		1.7
							*TRAILR1‐*397 T/G (TT vs TG + GG)	TG + CG genotype ↑ Recurrence		5.19
							*ICAM1* K469E (AA+AG vs GG)	GG genotype ↑ Recurrence		2.47
J2 [90]	2012	200 [Non‐treated (71), BCG (78)]	Ta (64), T1 (85), T2 (51)	G1 (67), G2 (43), G3 (90)	Recurrence (65), Non‐Recurrence (83)	iBCG + mBCG	Survivin 31G>C (GC vs GG)	GG genotype ↑ Recurrence	RFS	0.35
							Survivin 31G>C (CC vs GG)			0.22
I1 [91]	2011	99 [BCG (69), BCG + IFN‐α (30)	Cis (30), Ta (21), T1 (48), HC (85)	G1 (20), G2 (46) and G3 (33)	–	iBCG + mBCG	*NRAMP1* (GA vs GG)	GG genotype ↑ Recurrence	RFS	4.57
							*NRAMP1* (GT)n allele 3	↓ Recurrence		24.789
H6 [38]	2010	200 BlCa 265 Controls, BCG (78), Non‐treated (71)	Ta (64), T1 (85), T2 (51)	G1 (64), G2 (43), G3 (90)	Recurrence (65), Non‐Recurrence (84)	iBCG + mBCG	*p53R72P* G>C (GC vs GG)	GG genotype ↑ Recurrence	RFS	0.29
3.3. Metabolite										
X4 [92]	2025	Discovery Cohort 23	Cis (3), Ta (13), T1 (7)	LG (11), HG (12)	–	iBCG + mBCG	Threonine N6,N6,N6‐Trimethyl‐lysine Myristicacid Methioninesulfoxide Asymmetricdimethylarginine Guanidoacetic acid Choline 1‐Methyl‐4‐imidazoleacetic acid N‐Acetylneuraminic acid Creatinine Citrulline Argininosuccinic acid γ‐Glu‐Gly γ‐Glu‐Val Octanoylcarnitine S‐Methylcysteine‐S‐oxide	–	–	–
		Validation Cohort 40	Cis (6), Ta (10), T1 (24)	LG (5), HG (35)			Carnitine, Indole‐3‐Acetic acid, Valeric acid		RFS	6.13
4. Blood and Urine										
4.1 Protein										
O1 [27]	2017	PBL Group 51	Cis, Ta, T1	–	Recurrence (28), Non‐Recurrence (23)	–	IL‐8	↑ BCG Failure	RFS	4.24
		Urine Group 112	Cis/Ta (49), T1 (63)		Recurrence (48), Non‐Recurrence (64)					4.25
4.2 RNA										
T2 [25]	2022	204	T1 (204)	LG (16), HG (170)	Recurrence (16), Non‐Recurrence (88)	iBCG + mBCG	IL‐10	↓ BCG Response	Recurrence	15.4
									Progression	15.4
							CTLA‐4		Recurrence	3.18
									Progression	3.18
							FOXP3+		Recurrence	1.89
							T‐bet+		Recurrence	0.39
									Progression	0.39
							GATA3/T‐bet ratio		Recurrence	4.9
									Progression	1.6
5. Urine										
5.1 Protein										
T4 [28]	2022	64	Cis (5), Ta (30), T1 (29)	HG (64)	Recurrence (11), Non‐Recurrence (53)	–	A1AT	↑ Recurrence	RFS	–
							APOE			–
							ANG			42.89
							CA9			3.44
							IL‐8			–
							MMP9			–
							MMP10			3.86
							PAI1			–
							SDC1			–
							VEGFA			–
							Panel			–
J1 [26]	2012	72	Ta (49) and T1 (23), Controls (49)	G1 (8), G2 (26) and G3 (38)	–	iBCG	IL‐6/IL‐10 ratio	↑ Recurrence	RFS	4.09
D1 [39]	2003	20	–	–	–	iBCG	IL‐2	↓ Non‐Responder	–	0.368
5.2 DNA										
S2 [31]	2021	123	Cis (17), Ta (67), T1 (39)	LG (37), HG (86)	Recurrence (63)	iBCG iBCG + mBCG	Mycobacterium tuberculosis complex	↑ Recurrence/Progression	RFS	1 yr (0.967), 5 yr (0.817)
									PFS	0.942
6. Bladder washing										
6.1 Glycan and DNA										
A1 [93]	1987	66	–	–	–	iBCG	ABH reactivity, DNA ploidy	Favourable Prognosis	RFS PFS	–

**Table 2:** Information regarding predictive studies on bladder cancer patients treated with Bacillus Calmette‐Guérin. AUC, area under the curve; BCG, Bacillus Calmette‐Guérin; Cis, carcinoma *in situ*; G1, grade 1; G2, grade 2; G3, grade 3; HG, high‐grade; iBCG, induction BCG scheme; LG, low‐grade; mBCG, maintenance BCG scheme.

Study (ref)	Year	Cohort	Stage (*n*)	Histologic grade (*n*)	Outcome (*n*)	Treatment scheme	Molecule	Level of molecule	Sensitivity %	Specificity %	AUC
1. Tissue											
1.1 Protein											
X6 [51]	2025	73	Cis (73)	–	–	iBCG + mBCG	AR	↑ BCG Failure	81.2%	87.8%	0.905
							ADAR2		68.7%	85.4%	0.732
							PD‐L1		84.4%	87.8%	0.872
U3 [94]	2023	32	Cis (2), Ta (6), T1 (24)	LG (6), HG (26)	Responders (21), Non‐Responders (11)	iBCG + mBCG	GATA‐3/T‐bet ratio	↑ Response	91%	57%	0.74
Q1 [59]	2019	22	T1	HG	Responders (12), Non‐Responders (10)	iBCG + mBCG	Peritumoral T bet+/Lymphocyte	↑ Recurrence	75%	80%	–
							GTR/NLR	↓ Recurrence	75%	80%	
							Peritumoral T bet+	↑ Recurrence	66.7%	70%	
P4 [95]	2018	105	Cis (26), Ta (23), T1 (35), T1 + Concomitant Cis (21)	LG (32), HG (73)	Responders (89), Non‐Responders (16)	iBCG + mBCG	*NEDD9*	↑ Recurrence/Progression	83.6%	64.2%	–
									91.3%	51.2%	
								↓ BCG Response	84.1%	80.9%	
O2 [62]	2017	34	T1	G3	Recurrence (18), Progression (17)	iBCG + mBCG	*HER‐2*	↓ Recurrence/Progression	58%	83%	–
									65%	65%	
N1 [22]	2016	40	Cis (10), Ta (9) and T1 (21)	LG (7) and HG (33)	–	–	CD163+ TAM density	–	–	–	1
							CD68+ TAM density	–	–	–	1
							Total FOXP3+ cell density	–	–	–	1
							CD25+ cell density	–	–	–	0.92
							GATA3+ T‐cell density	–	–	–	0.99
							Total T‐bet+ T‐cell density	–	–	–	0.72
							CD4+ T‐cell count	–	–	–	0.8
L4 [96]	2014	38	Cis (38)	–	Responders (20), Non‐Responders (18)	iBCG	Ratio of GATA‐3+ lymphocytes/ T‐bet+ lymphocytes	↑ Responders	100%	80%	–
1.2 DNA											
R5 [32]	2020	Discovery Cohort 53	Cis, Ta, T1	HG (48), Not Specified (5)	Responders (26), Failures (27)	–	–	↑ BCG failure	–	–	–
		Internal Validation Cohort 37			Responders (19), Failures (18)		*KLF8, C12orf42, FLT1, WDR44, CHST11*				0.633 to 68.1
							*GPR158*				0.809
2. Blood											
2.1 Protein											
R2 [85]	2020	Discovery Cohort 13	T1 (50)	HG (9), LG (4)	Responders (9), Non‐Responders (4)	iBCG + mBCG	CCL27	–	–	–	–
		Validation Cohort 37		HG (24), LG (13)	Responders (28), Non‐Responders (9)			↑ BCG Response	Pre‐BCG treatment (67%)	Pre‐BCG treatment (78%)	Pre‐BCG treatment (0.730)
									Post‐BCG treatment (89%)	Post‐BCG treatment (68%)	Post‐BCG treatment (0.726)
									–	–	Combined timepoints GSCCL27 (0.897)
2.2 DNA											
M3 [37]	2015	204	Ta (82), T1 (122), Concomitant Cis (186)	LG (55), HG (149)	BCG Success (134), BCG Failure (70)	iBCG+ mBCG	*TNF‐α*1031T/C (TT + TC vs CC)	CC genotype ↑ Recurrence	–	–	0.82
							*IL4‐*33 T/C (CT vs TT)	–			
							*IL2RA* T/C (TT vs TC + CC)	TT genotype ↑ Recurrence			
							*IL17A*‐197G/A (GG + GA vs AA)	AA genotype ↑ Recurrence			
							*IL17RA*‐809A/G (AA+AG vs GG)	–			
							*IL18R1* rs3771171 T/C (CC vs TC + TT)	–			
							*FASL*‐844 T/C (TT + TC vs CC)	–			
							*TRAILR1*‐397 T/G (TT vs TG + GG)	TG + CG genotype ↑ Recurrence			
							*ICAM1* K469E (AA+AG vs GG)	GG genotype ↑ Recurrence			
3. Urine											
3.1 Protein											
T4 [28]	2022	64	Cis (5), Ta (30), T1 (29)	HG (64)	Recurrence (11), Non‐Recurrence (53)	–	A1AT	↑ Recurrence	81.8%	56.6%	0.6364
							APOE		81.8%	50.9%	0.6724
							ANG		63.6%	81.1%	0.7444
							CA9		63.6%	69.8%	0.6878
							IL‐8		36.4%	77.4%	0.5146
							MMP9		90.9%	26.4%	0.5420
							MMP10		72.7%	67.9%	0.7238
							PAI1		63.6%	66.0%	0.6261
							SDC1		54.5%	88.7%	0.6106
							VEGFA		27.3%	98.1%	0.5935
							Panel		81.8%	84.9%	0.8971
N3 [29]	2016	130	Ta (62), T1 (61)	LG (34), HG (96)	Recurrence (44), No Recurrence (86)	iBCG + mBCG	Panel: IL‐2, IL‐8, IL‐6, IL‐1ra, IL‐10, IL‐12[p70], IL‐12[p40], TRAIL, and TNF‐a	–	80%	77.4%	0.86
3.2 DNA											
X1 [30]	2025	65	Cis (9), Ta (31), T1 (25)	LG (4), HG (61)	BCG Failure (11)	iBCG + mBCG	Bladder Epicheck – DNA methylation	–	88%	92%	0.952
S2 [31]	2021	123	Cis (17), Ta (67), T1 (39)	LG (37), HG (86)	Recurrence (63)	iBCG iBCG + mBCG	Mycobacterium tuberculosis complex	↑ Recurrence	75%	100%	0.868
								↑ Progression	83.3%	91.5%	0.875

### 3.2 Quality assessment

**Fig. 2:**
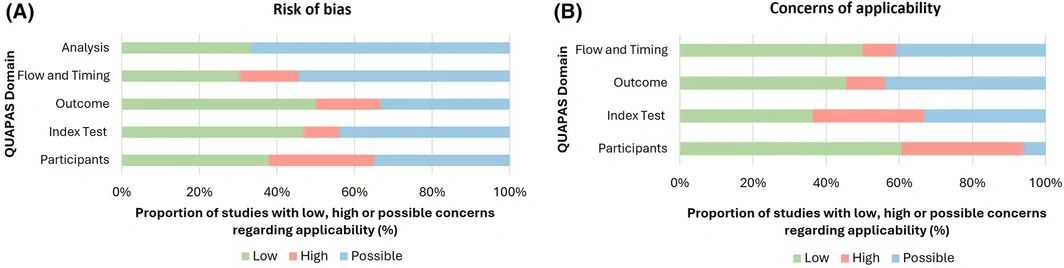
Quality assessment of the included prognostic studies using the quality assessment of prognostic accuracy studies (QUAPAS) tool. (A) Graphical illustration depicting the risk of bias in the prognostic studies. (B) Graphical illustration depicting the risk of concerns regarding the applicability of the prognostic studies.

**Fig. 3:**
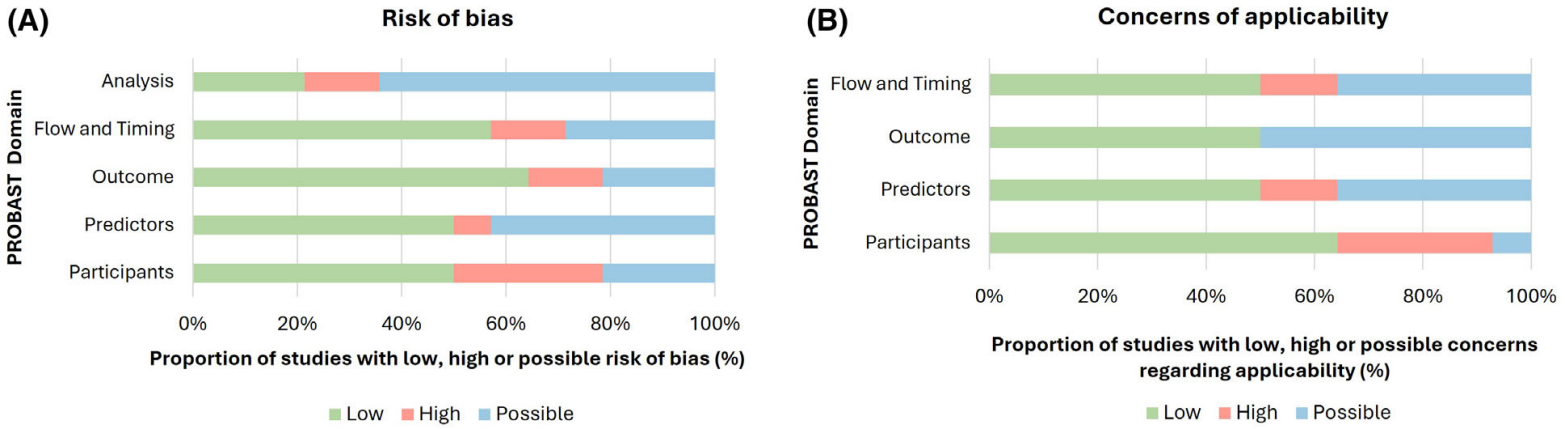
Quality assessment of the included predictive studies using the prediction model risk of bias assessment (PROBAST) tool. (A) Graphical illustration depicting the risk of bias in the predictive studies. (B) Graphical illustration depicting the risk of concerns regarding applicability in the predictive studies.

The QUAPAS and PROBAST tools were utilised to evaluate the quality of each study for prognostic and predictive studies, respectively (Fig. 2 and Table 3; Fig. 3 and Table 4). The signalling questions were tailored according to the objectives and were answered for each study (Tables S1 and S2). Regarding prognostic markers, most studies disclosed a low or possible risk of bias, as many signalling questions in the manuscripts could not be definitively addressed. This was especially prominent in the analysis domain, where 67% of the manuscripts showed a potential risk of bias, since it was challenging to determine whether information was missing or, in cases of data censoring, if the appropriate methods had ultimately been applied (Fig. 2A). Another domain associated with a high risk of bias, evident in 27% of the studies, related to participant selection (Fig. 2A). This risk arose mainly from the lack of random or consecutive patient sampling and the incomplete description of participant eligibility criteria. Regarding applicability concerns, the participants' domain had the highest rate of concern (33%). This was primarily due to the exclusion of important BlCa stages from the records, particularly patients with Cis or concomitant Cis (Fig. 2B). For predictive studies, applicability concerns and risk of bias in the participants' domain mirrored those seen in prognostic studies, with 29% depicting a high risk of bias (Fig. 3A,B).

**Table 3:** Information regarding risk of bias percentages of the included records regarding prognostic studies.

Risk of bias	Low (%)	High (%)	Possible (%)
Participants	38	27	35
Predictors	47	9	44
Outcome	50	17	33
Flow and timing	30	15	55
Analysis	33	0	67

**Table 4:** Information about risk of bias percentages of the included records, regarding predictive studies.

Risk of bias	Low (%)	High (%)	Possible (%)
Participants	50	29	21
Predictors	50	7	43
Outcome	64	14	21
Flow and timing	57	14	29
Analysis	21	14	64

### 3.3 Prognostic biomarkers

**Fig. 4:**
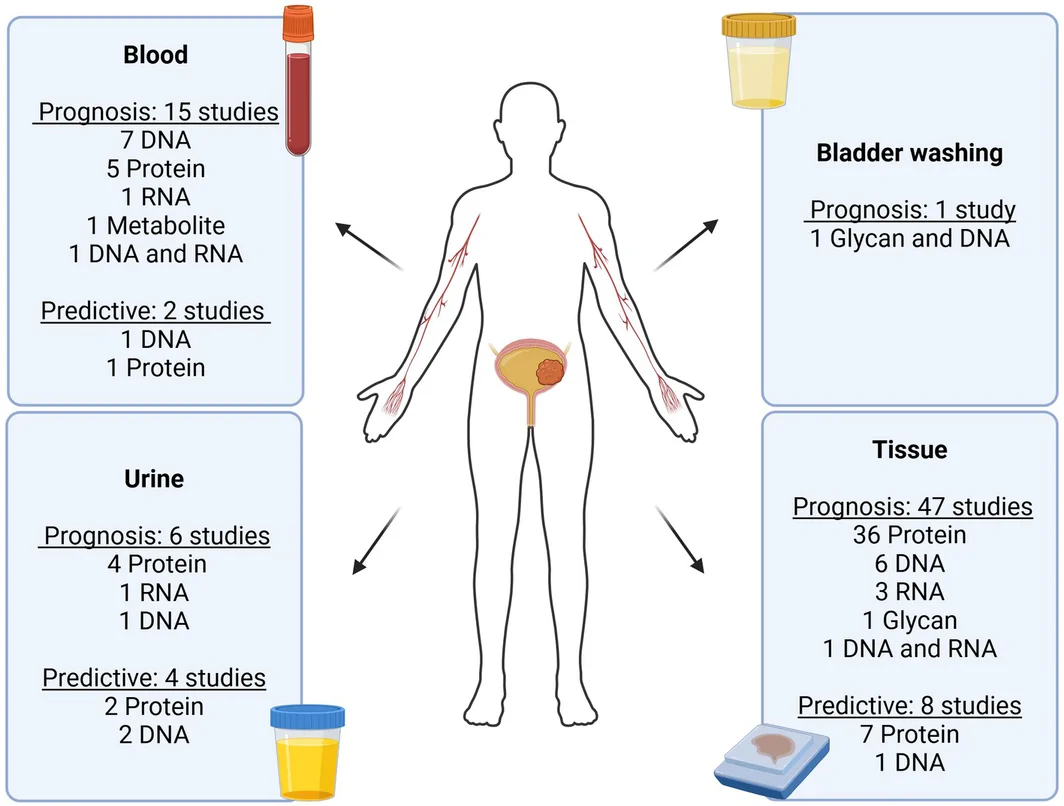
Biomarker study distribution by sample source and biomolecule type for Bacillus Calmette‐Guérin (BCG) failure. Figure created with BioRender.com.

Regarding the biomarker biological sources, 47 studies used tissues [mostly formalin‐fixed paraffin‐embedded (FFPE) tissues], 15 assessed blood, and 7 research articles explored urine (Fig. 4 and Table 1). Interestingly, most studies evaluated protein biomarkers, followed by deoxyribonucleic acid (DNA), ribonucleic acid (RNA), and glycans. Regarding tissue and urine studies, the most explored molecules were proteins, while DNA was the most explored type of biomarker in blood.

Concerning studies exploring tissue‐based biomarkers, 5 assessed the potential of protein 53 (p53) in tissue using immunohistochemistry [15, 16, 17, 18, 19], revealing this protein as an independent factor of progression‐free survival (PFS), disease‐specific survival (DSS), and/or recurrence‐free survival (RFS) in a multivariable analysis of BCG‐treated patients. In this regard, 2 studies associated p53 downregulation with BCG non‐response, while one linked p53 downregulation with disease recurrence [15, 16, 18]. Conversely, 2 other studies found p53 upregulation to be associated with both progression and recurrence [17, 19]. Additionally, 2 manuscripts examined the potential of Kiel 67 (Ki‐67) as a prognostic biomarker. Asakura et al [20] and Bertz et al. [21] found that increased Ki‐67 expression was associated with increased likelihood of cancer recurrence and progression. Although the cohorts differed between the two studies, as Asakura et al. [20] included patients with Ta G1 disease in addition to T1 tumours, while Bertz et al. [21] focused exclusively on patients with T1 disease with or without Cis, both reported similar trends of biomarker expression. Two additional reports evaluated the behaviour of cluster of differentiation 4 (CD4) in response to BCG treatment; however, the limited sample size and the substantial methodological differences preclude direct comparison of their findings. Moreover, Pichler et al. [22] demonstrated that a high CD4/CD8 ratio immunostaining was associated with a higher RFS, whereas Lim et al. [23] observed the same trend using CD4^+^/forkhead box P3^−^ (FOXP3) ratio analysed by immunofluorescence, which was higher in responders and also better predicted RFS after BCG treatment.

Moreover, Fukumoto and colleagues found that programmed cell death protein 1 (PD‐1) was an indicator of worse clinical outcome, with high PD‐1 expression in BCG‐relapsing tumours independently associating with subsequent tumour recurrence (RFS HR = 2.99), also constituting an independent risk factor for stage progression with a high HR (PFS HR = 8.27) [24].

A limited number of studies focused on biomarkers analysed in liquid biopsies such as urine or blood, with a high number of articles studying interleukins (IL). In a cohort of 204 T1 patients, Elsawy and colleagues showed that decreased IL‐10 levels in urine could reliably forecast recurrence and progression following BCG treatment [25]. Similarly, in 2012, Cai et al. analysed pre‐TURBT urine samples and found that a high IL‐6/IL‐10 ratio was associated with increased recurrence, with an RFS of 4.09 [26]. Furthermore, Qu et al found that higher levels of IL‐8 protein in urine and peripheral blood leucocytes were significantly associated with increased risk of recurrence compared to patients with lower IL‐8 levels [27].

### 3.4 Predictive biomarkers

Concerning studies which evaluated predictive biomarkers, 8 focused on tissue‐based markers, whereas 6 investigated liquid biopsies, specifically using urine or blood. Among those studies, most explored proteins, followed by DNA (Fig. 4 and Table 2). Protein‐based biomarkers AUCs ranged 0.64–1, with the maximum value reported for the proteins CD163, CD68, and FOXP3 analysed in FFPE tissues using immunohistochemistry [22]. Moreover, the Oncuria™ test was assessed for recurrence prediction in pre‐BCG treatment urines [28]. This test used a multiplex immunoassay to measure the levels of matrix metallopeptidase 9 (MMP9), vascular endothelial growth factor A (VEGFA), carbonic anhydrase 9 (CA9), syndecan‐1 (SDC1), plasminogen activator inhibitor 1 (PAI1), apolipoprotein E (APOE), alpha‐1 antitrypsin (A1AT), angiogenin (ANG), and matrix metallopeptidase 10 (MMP10), achieving a promising discriminatory performance with an AUC of 0.89, depicting 81.8% sensitivity and 84.9% specificity [28]. In the CyPRIT study, a nomogram score was created based on the score of changes in urine samples collected before BCG of 9 cytokines: IL‐2, IL‐8, IL‐6, IL‐1ra, IL‐10, IL‐12(p70), IL‐12(p40), tumour necrosis factor‐related apoptosis‐inducing ligand (TRAIL), and tumour necrosis factor‐alpha (TNF‐α) predicted recurrence in patients under BCG treatment, with an AUC of 0.855, 80% sensitivity and 77.4% specificity [29]. Besides the nomogram development, CyPRIT evaluated 130 Ta and T1 patients in similar proportions. As for studies focused on DNA‐based biomarkers, AUCs ranged from 0.63 to 0.952. Particularly noteworthy was the study of Roldán et al., testing Bladder Epicheck, a DNA methylation‐based test performed in urine sediment (CE approved for BlCa surveillance) as a predictive biomarker of BCG failure, with an estimated 88% sensitivity and 92% specificity (AUC of 0.952) [30]. Furthermore, a study by Muto et al. evaluated *Mycobacterium tuberculosis* complex DNA using polymerase chain reaction (PCR) in pre‐treatment urine samples, showing promise for predicting recurrence (AUC = 0.868) and progression (AUC = 0.875) [31]. Finally, Ilijazi et al. identified and validated G protein‐coupled receptor 58 (*GPR58*), krüppel‐like factor 8 (*KLF8*), chromosome 12 open reading frame 42 (*C12orf42*), WD repeat‐containing protein 44 (*WDR44*), vascular endothelial growth factor receptor 1 (*FLT1*), and carbohydrate sulfotransferase 11 (*CHST11*) promoter hypermethylation in FFPE tissues, finding that *GPR58* was the most promising biomarker, achieving an AUC of 0.809 for prediction of BCG failure [32].

## 4 Discussion

BCG therapy is commonly prescribed to patients with intermediate and high‐risk NMIBC [6, 7]. Although it constitutes the first immunotherapy in history used for cancer treatment, its mechanism of action remains largely unexplored [33]. Consequently, biomarkers enabling the precise identification of patients that might benefit from this therapy remain an unmet clinical need. Indeed, to this date, no FDA/CE‐approved prognostic or predictive biomarkers aiming to assess the outcome of these patients exist, notwithstanding multiple studies reporting on putative candidates. Thus, we aimed to systematically review and critically assess published studies that explored the potential of biomarkers for prognostic assessment and prediction of failure in BlCa patients treated with BCG therapy.

An extensive analysis of the publications identified a considerable number of studies investigating prognostic biomarkers, whereas studies assessing predictive biomarkers of BCG failure in BlCa were comparatively scarce. Most studies focused on protein markers, predominantly assessed in FFPE tissues. Among these, P53, Ki‐67 and pRB were evaluated in more than one study [15, 16, 17, 18, 19, 21, 34, 35, 36]. However, p53 expression was inconsistent across studies, likely reflecting differences in patients' cohort composition, as well as variability in the thresholds and protocols used for p53 assessment [15, 16, 17, 18, 19, 21, 34, 35, 36] (Fig. S1). In contrast, 2 independent studies consistently demonstrated the potential value of Ki‐67 for predicting recurrence, although further validation in larger and independent cohorts remains necessary to establish its clinical utility. Finally, pRB expression was investigated as a prognostic marker by Cormio et al. in 2 separate studies, evaluating its association with PFS and RFS, as well as its combined value with p53 for predicting PFS, reaching statistically significant results in both cases. Nevertheless, these findings were derived from a small cohort (*n* = 27), limiting the statistical power and generalisability of the results. Consequently, further validation in larger, independent patient cohorts is also required before pRB, either alone or in combination with p53, may be considered a reliable biomarker for predicting response to BCG therapy. In the same vein, some biomolecules were also assessed in more than one biological source. For instance, FAS Ligand (FASL), FOXP3, GATA3 and cytotoxic T‐lymphocyte associated protein 4 (CTLA‐4) were evaluated in tissue and blood, whereas IL‐2, IL‐8 and IL‐10 were studied in blood and urine [15, 16, 17, 18, 19, 23, 25, 26, 27, 28, 37, 38, 39] (Fig. S1), and MMP9 in tissue and urine [28, 40] (Fig. S1). However, direct comparison between these studies remains challenging owing to differences in biological specimens, analytical methodologies, and biomarker quantification protocols. Nonetheless, these findings suggests that those biomarkers might be closely related with the mechanism of action of BCG treatment and further emphasises their clinical potential. As expected, we found that a significant number of the studied biomarkers were related to the immune system, including interleukins, immune cell markers, and immune checkpoint molecules such as PD‐1 and CTLA‐4. Indeed, because BCG instillation is an immunotherapy, immunological factors are likely to play a significant role in therapeutic success or failure. It is, thus, anticipated that immune factors associated with an effective immune response might be highly activated and identify patients more likely to respond to BCG treatment [41, 42].

Driven by the morbidity and invasive nature of cystoscopy allied to the low sensitivity of urine cytology for BlCa detection as well as follow‐up, liquid biopsies emerge as an alternative for those limiting strategies [43]. Importantly, it is acknowledged that liquid biopsies, such as urine and blood, which are collected using non‐ or minimally invasive means, might better represent tumour and microenvironment heterogeneity compared with tissues [44], allied to the fact that they allow real‐time monitoring of molecular features of the disease. In the context of BlCa, urine is particularly important since it comes into direct contact with tumour cells and their microenvironment, eventually providing a more comprehensive analysis of prognostic and predictive markers of BCG treatment failure [43]. Still, liquid biopsies encompass hurdles such as detecting molecules in disease with low molecular burden, lack of technical standardisation as well as hampered validation for clinical usage [45]. Notwithstanding these limitations concerning liquid biopsies, the CyPRIT clinical trial (NCT01007058, completed in 2016) involved the evaluation of a panel of 9 cytokines in urine supernatant for prediction of recurrence in patients undergoing BCG therapy, having reached promising results (AUC = 0.855) [29] in a large cohort of patients (*n* = 140). Importantly, the CyPRITs' nomogram, based on cytokine scores, facilitates clinical interpretation by estimating an individual's risk of recurrence, representing a promising tool for patient management. Nevertheless, despite these encouraging findings, this biomarker panel has not been externally validated in large independent multicentre cohorts yet, limiting translation into routine clinical practice. Additionally, Bladder Epicheck^®^, a urine test based on DNA methylation (CE/FDA approved for BlCa surveillance) [46] was tested by Roldán et al. for BCG failure prediction. While tested in a small cohort of patients (*n* = 65), this test obtained promising results due to its high AUC value (0.95) [30]. Although FDA/CE approval has not been granted for this purpose, Bladder Epicheck® is CE approved for BlCa surveillance, which might facilitate its clinical implementation and approval for BCG failure prediction after successful validation in larger patient cohorts. Remarkably, a test consisting of a panel of proteins detected in urine which also predicts BCG failure – Oncuria™ – has earned FDA's Breakthrough Device Designation [28]. Although this designation highlights its potential clinical value, it does not imply full FDA approval, and further validation in large independent cohorts is required before routine implementation in clinical practice is considered. These tests might be clinically useful for the management of BCG patients if incorporated within future AUA/EAU guidelines after FDA/CE approval and might aid in treatment decisions before starting BCG by predicting which patients may not benefit from this treatment. These patients would benefit from enrolment in clinical trials or be proposed for cystectomy, depending on tumour histopathological features and patients' individual conditions.

Importantly, several drawbacks were apparent in the studies analysed. One major concern pertains to applicability because a significant number of studies inadequately reported the variables of interest, hampering the reproducibility of the tests reported. Additionally, most studies reported their findings based on small patient cohorts, potentially introducing bias and lacking representativeness. Another important limitation, which may represent a major barrier for the clinical translation of promising multi‐marker panels such as Oncuria™ and CyPRIT, is the lack of independent validation in larger cohorts. This gap likely reflects the inherent challenges of retrospective and prospective biomarker research in the BCG setting, including the prolonged duration of BCG treatment and follow‐up, particularly for maintenance regimens that may last up to three years. This challenge may limit large‐scale validation studies, despite their essential role in establishing the robustness, clinical validity, and potential implementation of these biomarker assays.

Furthermore, we found a limited number of reports assessing predictive biomarkers, most of them preliminary, with the proposed biomarkers still requiring validation on larger and more robust independent cohorts. Moreover, a significant heterogeneity of the studies complicates direct inter‐study comparison and renders meta‐analysis unfeasible. Importantly, according to EAU guidelines, to achieve optimal BCG efficacy, it is recommended to administer an induction schedule (iBCG) consisting of 6‐weekly instillations aimed at activating the initial innate and adaptive immune response against residual tumour cells, followed by a maintenance schedule (mBCG) from 1 year up to 3 years (depending on patients' risk) [47]. Notably, mBCG is intended to sustain long‐term anti‐tumour immune surveillance and thus reduce the risk of recurrence [47, 48, 49]. In this regard, the studies assessed in this systematic review revealed a significant variation in BCG administration schedules, with 56% of the studies involving patients who underwent both induction and maintenance. Indeed, this heterogeneity might influence patient outcomes and may impact biomarker profiles, since the immunological display and tumour microenvironment differ substantially between induction and maintenance schedules. The heterogeneity in BCG treatment schedules across the included studies and the available evidence do not allow for a direct comparison of biomarker expression or performance between iBCG and mBCG cohorts. Specifically, only a few studies evaluated the same biomarkers in both treatment regimens, precluding a meaningful direct comparison. Consequently, that analysis was not undertaken in this systematic review.

Although our work highlights several promising biomarkers, it is important to emphasise the need for further research to improve reliability, robustness and performance reproducibility before implementation in clinical practice as validated prognostic and/or predictive tools. Nevertheless, the evidence gathered in this systematic review provides a strong foundation for the development of multi‐marker panels integrating different biomolecule classes and sample sources. It also supports the testing of emerging biomarker categories, such as extracellular vesicles and small RNAs, which may further improve the accuracy of predicting response to BCG therapy. Considering the challenges of recruiting enough BCG‐treated patients within a single institution, the successful translation of biomarkers into clinical practice will heavily depend on collaborative, multicentre studies using standardised methodologies to ensure robust validation and facilitate clinical implementation.

## 5 Conclusions

In this systematic and comprehensive review, we found that a diverse range of biomolecules holding potential as prognostic and/or predictive biomarkers for failure of BCG therapy have been reported but seldom validated. These biomarkers might improve clinical management of BlCa patients, who are currently eligible for BCG treatment, based mostly on risk assessment of progression/recurrence but having a high likelihood of not responding to therapy, enduring its side effects without clinical benefit. These biomarkers are clearly and urgently needed to improve care and prognosis of BlCa patients.

## Conflict of interest

The authors declare that they have no competing interests.

## Author contributions

Conceptualisation, RR‐P and AT‐M; writing—RR‐P and AT‐M; writing—review and editing, RR‐P, AT‐M, RF, JAC, GIR, SM‐R, RH and CJ; supervision, SM‐R, RH and CJ. All authors have read and agreed to the published version of the manuscript.

## Supporting information


**Fig. S1.** Venn diagram of the studied biomarker molecules by source.
**Table S1.** Quality Assessment of each included study through quality assessment of prognosis accuracy studies (QUAPAS).
**Table S2.** Quality assessment of each included study through prediction model risk of bias assessment (PROBAST).
