# Supplementary material for: Predictive and prognostic biomarkers of Bacillus Calmette‐Guérin therapy failure in bladder cancer patients: A systematic review
Source: Mol Oncol. 2026 Sep 7:10.1002/1878-0261.70329. Online ahead of print. doi: 10.1002/1878-0261.70329 (PMC13548025; doi:10.1002/1878-0261.70329)
Supplement: Supplementary file 1 [file MOL2-9999-0-s001.docx]

**Supplementary Material:**

*Supplementary Tables*

**Table S1:** Quality Assessment of each included study through quality assessment of prognosis accuracy studies (QUAPAS).

| **Author, year, journal** | **Study ID** | **Risk of bias** | | | | | | | | | | | | | | | | | | | | | | **Concerns of applicability** | | | |
| --- | --- | --- | --- | --- | --- | --- | --- | --- | --- | --- | --- | --- | --- | --- | --- | --- | --- | --- | --- | --- | --- | --- | --- | --- | --- | --- | --- |
|  |  | **Participants** | | | | **Index test** | | | | | **Outcome** | | | | **Flow and timing** | | | | **Analysis** | | | | | **P** | **T** | **O** | **F** |
|  |  | **P1** | **P2** | **P3** | **P** | **T1** | **T2** | **T3** | **T4** | **T** | **O1** | **O2** | **O3** | **O** | **F1** | **F2** | **F3** | **F** | **A1** | **A2** | **A3** | **A4** | **A** |  |  |  |  |
| P. Deng et al, 2025, Gene | X7 | Y | U | U | Possible | N | Y | U | Y | Possible | Y | Y | U | Low | Y | U | U | Possible | U | U | U | U | Possible | Possible | Possible | Possible | Possible |
| G. Ricciardi et al, 2025, Lab Invest | X6 | Y | U | U | Possible | Y | Y | U | Y | Low | Y | Y | Y | Low | Y | U | U | Possible | N | U | U | U | Possible | High | Possible | Possible | Possible |
| T. Yamashita et al, 2025, Pathology International | X5 | Y | U | U | Possible | Y | Y | U | Y | Low | Y | Y | N | High | U | U | U | Possible | N | U | U | Y | Possible | Low | Low | Low | Possible |
| M. Miyake et al, 2025, IOS Press | X4 | Y | U | Y | Low | Y | Y | U | U | Possible | Y | Y | U | Low | N | Y | Y | Possible | Y | U | U | U | Possible | Low | Possible | Low | Low |
| M. Lima et al, 2025, Pathol – Research and Practice | X3 | Y | U | Y | Low | Y | Y | Y | Y | Low | Y | Y | Y | Low | N | Y | U | High | Y | U | U | U | Possible | Low | Low | Low | Possible |
| I Ruiz-Lorente, 2025, Biomedicines | X2 | Y | U | Y | Low | Y | Y | U | U | Possible | Y | Y | U | Low | U | Y | U | Possible | Y | U | U | U | Possible | Low | Low | Possible | Low |
| Q-D Xia et al, 2024, Precision Oncol | V3 | Y | U | Y | Low | Y | U | U | U | Possible | Y | Y | U | Low | U | Y | Y | Low | Y | U | U | U | Possible | Low | Low | Low | Low |
| L. Jin et al, 2024, Frontiers Immunol | V2 | Y | U | Y | Low | Y | U | U | U | Possible | Y | Y | U | Low | N | U | U | High | Y | U | U | U | Possible | Low | Possible | Low | High |
| L. Zucca et al, 2024, Trans Oncol | V1 | Y | U | Y | Low | Y | Y | U | U | Low | Y | Y | U | Low | Y | Y | Y | Low | Y | U | U | N | Possible | Low | Possible | Low | Low |
| W. Nam et al, 2023, Frontiers in Immunology | U4 | Y | U | Y | Low | Y | Y | N | U | Possible | Y | Y | U | Low | Y | U | Y | Low | Y | U | U | Y | Low | Low | Possible | Low | Low |
| R. Nishikawa et al, 2023, Inter J Urol | U2 | Y | U | Y | Low | Y | Y | N | Y | Low | Y | Y | U | Low | U | Y | U | Possible | Y | U | U | N | Possible | Low | Possible | Low | High |
| X. Tan et al, 2023, Eur Urol Oncol | U1 | Y | Y | Y | Low | Y | N | U | Y | Possible | Y | Y | U | Low | N | Y | Y | Possible | N | U | U | Y | Possible | Low | Low | Low | Low |
| X. M. Piao et al, 2022, Urol Oncology | T5 | Y | U | Y | Low | N | Y | N | U | High | Y | Y | U | Low | U | Y | U | High | Y | U | U | Y | Low | High | High | Possible | Low |
| K. Murakami et al, 2022, Cancer Biomarkers | T4 | U | U | Y | Possible | Y | Y | Y | U | Low | Y | Y | U | Low | U | Y | Y | Low | Y | U | U | Y | Low | Low | Low | Possible | Possible |
| A. Awadalla et al, 2022, Clin Genito Cancer | T3 | N | U | Y | High | Y | Y | U | Y | Low | Y | Y | U | Low | Y | Y | Y | Low | Y | U | U | Y | Low | Possible | Low | Possible | Low |
| AA. Elsawy et al, 2022, BJU Inter | T2 | Y | Y | Y | Low | Y | Y | U | U | Low | U | U | U | Possible | Y | Y | Y | Low | Y | U | U | Y | Low | Low | Possible | Low | Low |
| M. Kobayashi et al, 2022, Can Immuno, | T1 | Y | U | Y | Low | Y | Y | U | U | Low | Y | Y | U | Low | U | Y | U | Possible | Y | U | U | Y | Low | Low | High | Possible | Low |
| S. Muto et al, 2021, Euro Urol Open Science | S2 | Y | U | Y | Low | N | Y | N | N | High | U | Y | N | High | Y | U | Y | Possible | Y | U | U | U | Possible | Low | High | Possible | Low |
| J. Sanders et al, 2021, Transl Androl Urol | S1 | Y | N | Y | High | Y | Y | U | N | Possible | U | U | U | Possible | Y | Y | Y | Low | Y | U | U | U | Possible | Low | Possible | Possible | Low |
| D. A. Bastos et al, 2020, IOS Press | R4 | Y | Y | Y | Low | Y | Y | U | N | Possible | N | Y | N | High | Y | Y | Y | Low | Y | U | U | Y | Low | Low | High | Low | Low |
| R. Batista et al, 2020, Inter Journ Molec Sciences | R3 | N | U | Y | High | N | U | U | N | High | Y | U | U | Possible | Y | U | U | Possible | N | U | U | Y | Possible | Low | High | High | Possible |
| W. Zhong et al, 2020, OncoImmunology | R2 | N | Y | Y | High | Y | Y | Y | Y | Low | Y | Y | U | Low | Y | Y | Y | Low | Y | U | U | Y | Low | High | Low | Low | Low |
| CJ. Lim et al, 2020, Frontiers in Immunology | R1 | N | U | Y | High | Y | N | N | U | High | Y | N | U | High | Y | Y | U | Low | U | U | U | N | Possible | Low | High | High | High |
| R. Martínez et al, 2019, OncoImmunology | Q1 | N | U | Y | High | U | Y | U | Y | Low | Y | Y | N | High | Y | Y | Y | Low | Y | U | U | N | Possible | High | Possible | Low | Low |
| F. Sanguedolce et al, 2018, Jour Canc Rese Cli On | P3 | U | Y | Y | Low | Y | Y | U | Y | Low | Y | Y | Y | Low | Y | Y | Y | Low | Y | U | U | Y | Low | Low | Low | Low | Possible |
| K. Fukumoto et al, 2018, Ann Surg Oncol | P2 | Y | Y | N | High | Y | Y | U | Y | Low | Y | Y | U | Low | U | U | Y | Low | Y | U | U | Y | Low | Low | Low | Possible | Low |
| R. Mano et al, 2018, Urologic Oncology | P1 | Y | Y | Y | Low | Y | Y | Y | Y | Low | Y | Y | Y | Low | Y | Y | Y | Low | Y | U | U | Y | Low | Possible | Low | Possible | Possible |
| A. C. Dowell et al, 2017, Plos One | O4 | Y | N | Y | High | Y | Y | Y | U | Low | U | Y | U | Possible | U | Y | Y | Low | U | U | U | U | Possible | Possible | Possible | High | Possible |
| M. Miyake et al, 2017, Inter Journ Molec Sciences | O3 | Y | Y | Y | Low | Y | Y | U | Y | Low | U | Y | U | Possible | Y | Y | Y | Low | Y | U | U | Y | Low | Low | Low | Possible | Possible |
| L. Cormio et al, 2017, Oncotarget | O2 | Y | U | Y | Low | N | U | U | U | Possible | U | Y | U | Possible | Y | N | Y | Possible | N | U | U | Y | Possible | Low | Low | Low | Low |
| K. Qu et al, 2017, OncoImmunology | O1 | Y | Y | Y | Low | U | Y | U | N | High | Y | Y | U | Low | Y | Y | Y | Low | Y | U | U | N | Possible | Low | Possible | Possible | Possible |
| A. Mbeutcha et al, 2016, Urol Oncol | N2 | U | U | Y | Possible | N | Y | U | Y | Possible | Y | Y | U | Low | N | Y | U | Possible | N | U | U | Y | Possible | Low | High | Low | Low |
| R. Pichler, 2016 et al, Oncotarget | N1 | U | U | Y | Possible | N | U | U | U | Possible | U | U | U | Possible | U | Y | U | Possible | U | U | U | N | Possible | Low | High | Possible | Low |
| L. Lima et al, 2015, BJU Int | M3 | U | N | Y | High | N | Y | Y | U | Possible | Y | Y | U | Low | U | Y | Y | Possible | Y | U | U | Y | Low | Low | High | Low | Low |
| C. Ryk et al, 2015, Redox Biol | M2 | U | Y | Y | Low | U | Y | U | U | Possible | U | U | U | Possible | U | U | U | Possible | U | U | U | N | Possible | Low | Possible | Possible | Possible |
| JM. Gaya et al, 2015, J Urol | M1 | U | U | Y | Possible | N | Y | U | Y | Possible | Y | Y | U | Low | N | Y | U | Possible | N | U | U | Y | Possible | High | High | Low | Low |
| L. Lima et al, 2014, Urol Oncol | L3 | U | U | Y | Possible | U | Y | U | Y | Low | N | Y | U | High | Y | Y | Y | Low | Y | U | U | Y | Low | Low | Possible | Low | Low |
| L. Lima et al, 2014, Urol Oncol | L2 | U | N | Y | High | N | Y | Y | U | Possible | U | Y | U | Possible | N | Y | Y | High | N | U | U | Y | Possible | Low | High | Possible | Low |
| S. Bertz et al, 2014, Eur Urol | L1 | U | U | Y | Possible | U | Y | U | Y | Low | U | Y | U | Possible | U | U | U | Possible | U | U | U | Y | Possible | High | Possible | Possible | Possible |
| F. Ajili et al, 2013, Ultrastruct Pathol | K4 | Y | U | Y | Low | N | Y | U | U | Possible | U | Y | U | Possible | Y | Y | Y | Low | Y | U | U | Y | Low | Low | High | Possible | Low |
| L. Lima et al, 2013, Br J Cancer | K3 | U | U | Y | Possible | U | Y | U | U | Possible | U | U | U | Possible | Y | U | Y | Possible | Y | U | U | Y | Low | Low | Possible | Possible | Low |
| M. Oderda et al, 2013, Urol Int | K2 | U | Y | Y | Low | U | Y | U | Y | Low | Y | Y | U | Low | U | Y | U | Possible | U | U | U | Y | Possible | Low | Possible | Low | Low |
| F. Ajili et al, 2012, Ultrastruct Pathol | J4 | Y | Y | N | High | Y | Y | U | Y | Low | U | Y | U | Possible | Y | Y | U | Possible | U | U | U | Y | Possible | Low | Low | Possible | Low |
| F. Ajili et al, 2012, Ultrastruct Pathol | J3 | Y | Y | N | High | U | Y | U | Y | Low | N | Y | U | High | Y | Y | U | Possible | Y | U | U | Y | Low | High | Possible | High | Low |
| PK. Jaiswal et al, 2012, Mol Biol Rep | J2 | U | N | N | High | N | Y | U | U | Possible | U | U | U | Possible | U | U | U | Possible | U | U | U | Y | Possible | High | High | Possible | Possible |
| T. Cai et al, 2012, Exp Ther Med | J1 | Y | Y | N | High | Y | Y | U | Y | Low | N | Y | U | High | Y | Y | U | Possible | Y | U | U | Y | Low | High | Low | High | Low |
| S. Alkhateeb et al, 2011, Urol Ann | I4 | Y | U | Y | Low | Y | Y | U | Y | Low | Y | Y | U | Low | U | U | U | Possible | U | U | U | N | Possible | Low | Low | Low | High |
| W. Otto et al, 2011, J Urol | I3 | U | U | Y | Possible | U | Y | U | U | Possible | U | U | U | Possible | N | N | U | High | N | U | U | Y | Possible | High | Possible | Possible | Possible |
| M. Agundez et al, 2011, Eur Urol | I2 | U | U | Y | Possible | U | Y | U | U | Possible | U | U | U | Possible | U | U | U | Possible | U | U | U | Y | Possible | High | Possible | Possible | Possible |
| E. Chiong et al, 2011, Eur Urol | I1 | U | U | Y | Possible | N | Y | U | Y | Possible | Y | Y | U | Low | U | U | U | Possible | U | U | U | Y | Possible | Low | High | Low | Possible |
| P. Srivastava et al, 2010, Cancer Biomark | H6 | U | N | Y | High | N | Y | U | U | Possible | U | U | U | Possible | Y | U | Y | Possible | Y | U | U | N | Possible | High | High | Possible | Low |
| A. Lopez-Beltran et al, 2010, Virchows Arch | H5 | Y | U | Y | Low | Y | Y | U | Y | Low | U | U | U | Possible | Y | U | U | Possible | U | U | U | U | Possible | High | Low | Possible | Possible |
| M. Alvarez-Múgica et al, 2010, J Urol | H4 | U | U | Y | Possible | Y | Y | U | U | Low | U | U | U | Possible | N | U | U | Possible | N | U | U | Y | Possible | High | Low | High | Possible |
| T. Cai et al, 2010, J Urol | H3 | Y | U | N | High | N | Y | U | U | Possible | N | Y | U | High | U | U | U | Possible | U | U | U | Y | Possible | High | High | High | Possible |
| L. Cormio et al, 2010, Urol Oncol | H2 | U | U | Y | Possible | Y | Y | Y | Y | Low | Y | Y | U | Low | U | U | U | Possible | U | U | U | Y | Possible | High | Low | Low | Possible |
| YJ. Kim et al, 2010, Clin Cancer Res | H1 | U | U | Y | Possible | N | Y | U | U | Possible | Y | Y | U | Low | U | U | U | Possible | U | U | U | Y | Possible | High | High | Low | Possible |
| C. Ayari et al, 2009, Eur Urol | G3 | U | U | Y | Possible | N | Y | U | Y | Possible | U | U | U | Possible | N | U | U | Possible | Y | U | U | Y | Low | Low | High | Possible | Possible |
| J. Palou et al, 2009, Eur Urol | G2 | U | U | Y | Possible | Y | Y | U | Y | Low | U | N | U | High | Y | N | U | High | Y | U | U | Y | Low | High | Low | Possible | High |
| L. Cormio et al, 2009, Anticancer Res | G1 | U | U | Y | Possible | Y | Y | U | Y | Low | Y | Y | U | Low | U | U | U | Possible | U | U | U | N | Possible | Low | Low | Low | Possible |
| JA. Queipo-Zaragoza et al, 2007, Scand J Urol Nephrol | F1 | U | U | Y | Possible | Y | Y | Y | Y | Low | U | U | U | Possible | Y | Y | N | High | Y | Y | U | Y | Low | High | Low | Possible | Low |
| F. Saint et al, 2004, Eur Urol | E1 | Y | U | Y | Low | N | Y | U | U | Possible | Y | Y | U | Low | Y | U | Y | Possible | U | U | U | Y | Possible | Low | High | Low | Low |
| E. Watanabe et al, 2003, Cancer Immunol Immunother | D1 | U | U | Y | Possible | Y | Y | U | U | Low | Y | Y | U | Low | U | U | U | Possible | U | U | U | Y | Possible | High | Possible | Low | Possible |
| T. Asakura et al, 1997, Eur Urol | C2 | U | U | N | High | N | Y | U | Y | Possible | Y | Y | U | Low | Y | N | Y | High | Y | U | U | Y | Low | High | Low | Low | High |
| E. Lee et al, 1997, Int J Urol | C1 | U | U | Y | Possible | N | Y | U | Y | Possible | N | Y | U | High | U | Y | Y | Low | U | U | U | Y | Possible | High | High | Low | Low |
| L. Lacombe et al, 1996, J Clin Oncol | B1 | U | U | Y | Possible | N | Y | N | Y | High | U | U | U | Possible | N | U | N | High | U | U | U | Y | Possible | Low | Low | Possible | Possible |
| E. Orihuela et al, 1987, Cancer | A1 | U | N | Y | High | N | Y | Y | Y | Low | Y | Y | U | Low | N | N | U | High | N | U | U | Y | Possible | Low | Possible | Low | Possible |
| QUAPAS items: P1= Participant selection is fully described; P2= A consecutive or random sample of patients was selected; P3= A case-control design was avoided; P= Risk of Bias on Participants; T1= The index test was well described; T2= Method for performing the index test was the same for all the participants; T3= The index test results were interpreted without knowledge of the outcome; T4= The threshold used was pre-specified; T= Risk of Bias on Index Test; O1= The Method used to measure the outcome was valid and reliable (only if reported a histological sample); O2= The method for measuring the outcome was the same for all participants; O3= The outcome was measured without knowledge of the index test results; O= Risk of Bias on Outcome; F1= Description of patients lost-to-follow up; F2= Appropriate interval between index and outcome; F3= All the patients outcome is available; F= Risk of Bias on Flow and Timming; A1= All the participants were enrolled in the analysis; A2= Data missing, the correct methods were used; A3= Appropriate methods used accounting censoring; A4= Competing events appropriate methods were used to account them; A= Risk of Bias on Analysis; Y= Fulfilled; N= Not fulfilled; U= Unclear; It was considered a minimum follow-up time of 2 years. | | | | | | | | | | | | | | | | | | | | | | | | | | | |

**Table S2:** Quality assessment of each included study through prediction model risk of bias assessment (PROBAST).

| **Author, year, journal** | **Study ID** | **Risk of bias** | | | | | | | | | | | | | | | | | | | | | | | **Concerns of applicability** | | | |
| --- | --- | --- | --- | --- | --- | --- | --- | --- | --- | --- | --- | --- | --- | --- | --- | --- | --- | --- | --- | --- | --- | --- | --- | --- | --- | --- | --- | --- |
|  |  | **Participants** | | | | **Predictors** | | | | | **Outcome** | | | | **Flow and Timing** | | | | **Analysis** | | | | | | **P** | **T** | **O** | **F** |
|  |  | **P1** | **P2** | **P3** | **P** | **T1** | **T2** | **T3** | **T4** | **T** | **O1** | **O2** | **O3** | **O** | **F1** | **F2** | **F3** | **F** | **A1** | **A2** | **A3** | **A4** | **A5** | **A** |  |  |  |  |
| G. Ricciardi et al, 2025, Lab Invest | X6 | Y | U | U | Possible | Y | Y | U | Y | Low | Y | Y | Y | Low | Y | U | U | Possible | N | U | U | U | Y | Possible | Possible | Possible | Possible | Possible |
| F. L. Roldan et al, 2025, BJU International | X1 | Y | U | Y | Low | U | Y | Y | U | Low | Y | Y | U | Low | N | U | N | High | U | U | U | U | Y | Possible | Low | Possible | Low | High |
| G. M. Villoldo et al, 2023, Tech Science Press | U3 | Y | Y | Y | Low | Y | Y | U | Y | Low | Y | Y | U | Low | Y | Y | Y | Low | Y | U | U | N | U | Possible | Low | Low | Low | Low |
| K. Murakami et al, 2022, Cancer Biomarkers | T4 | U | U | Y | Possible | Y | Y | Y | U | Low | Y | Y | U | Low | U | Y | Y | Low | Y | U | U | U | Y | Low | Low | Low | Possible | Possible |
| S. Muto et al, 2021, Euro Urol Open Science | S2 | Y | U | Y | Low | N | Y | N | N | High | U | Y | N | High | Y | U | Y | Low | Y | U | U | N | U | Possible | Low | High | Possible | Low |
| W. Zhong et al, 2020, OncoImmunology | R2 | N | Y | Y | High | Y | Y | Y | Y | Low | Y | Y | U | Low | Y | Y | Y | Low | Y | U | U | U | Y | Low | High | Low | Low | Low |
| D. Ilijazi et al, 2020, Cells | R5 | Y | Y | Y | Low | Y | U | U | U | Possible | Y | Y | U | Low | Y | Y | Y | Low | Y | U | U | Y | U | Low | Low | Possible | Possible | Low |
| R. Martínez et al, 2019, OncoImmunology | Q1 | N | U | Y | High | U | Y | U | Y | Low | Y | Y | N | High | Y | Y | Y | Low | Y | U | U | N | U | Possible | High | Possible | Low | Low |
| I. M. El-Babouly et al, 2018, Urologic Oncology | P4 | Y | Y | Y | Low | Y | Y | Y | Y | Low | U | Y | Y | Low | Y | Y | Y | Low | Y | U | U | N | Y | Possible | Low | Low | Possible | Possible |
| L. Cormio et al, 2017, Oncotarget | O2 | Y | U | Y | Low | N | U | U | U | Possible | U | Y | U | Possible | Y | N | Y | Possible | N | U | U | Y | Y | Possible | Low | Low | Low | Low |
| A. M. Kamat et al, 2016, European Urology | N3 | Y | U | Y | Low | N | Y | U | U | Possible | U | U | U | Possible | U | U | Y | Possible | Y | U | U | N | Y | Possible | Low | Low | Possible | Possible |
| R. Pichler et al, 2016, Oncotarget | N1 | U | U | Y | Possible | N | U | U | U | Possible | U | U | U | Possible | U | Y | U | Possible | U | U | U | N | N | High | Low | High | Possible | Low |
| L. Lima et al, 2015, BJU International | M3 | U | N | Y | High | N | Y | Y | U | Possible | Y | Y | U | Low | U | Y | Y | Low | U | U | U | N | N | High | High | Low | Low | Possible |
| R. Nunez-Nateras et al, 2014 | L4 | Y | U | N | High | N | Y | U | Y | Possible | Y | Y | U | Low | Y | N | Y | High | U | U | U | N | Y | Possible | High | Possible | Low | High |
| PROBAST items: P1= Participant selection is fully described; P2= A consecutive or random sample of patients was selected; P3= A case-control design was avoided; P= Risk of Bias on Patient Selection; T1= The index test was well described; T2= Method for performing the index test was the same for all the participants; T3= The index test results were interpreted without knowledge of the outcome; T4= The threshold used was pre-specified; T= Risk of Bias on Index Test; O1= The Method used to measure the outcome was valid and reliable (only if reported a histological sample); O2= The method for measuring the outcome was the same for all participants; O3= The outcome was measured without knowledge of the index test results; O= Risk of Bias on Outcome; F1= Description of patients lost-to-follow up; F2= Appropriate interval between index and outcome; F3= All the patients outcome is available; F= Risk of Bias on Flow and Timming; A1= All the participants were enrolled in the analysis; A2= Data missing, the correct methods were used; A3= Appropriate methods used accounting censoring; A4= Complexities in the data were accounted for appropriately; A5= Relevant model performance measures were evaluated appropriately; A= Risk of Bias on Analysis; Y= Fulfilled; N= Not fulfilled; U= Unclear; It was considered a minimum follow-up time of 2 years. | | | | | | | | | | | | | | | | | | | | | | | | | | | | |

*Supplementary Figure*

**Figure S1:** Venn diagram of the studied biomarker molecules by source. Figure created with Biorender.com.
